# Circulating miR-17, miR-20a, miR-29c, and miR-223 Combined as Non-Invasive Biomarkers in Nasopharyngeal Carcinoma

**DOI:** 10.1371/journal.pone.0046367

**Published:** 2012-10-08

**Authors:** Xi Zeng, Juanjuan Xiang, Minghua Wu, Wei Xiong, Hailin Tang, Min Deng, Xiayu Li, Qianjin Liao, Bo Su, Zhaohui Luo, Yanhong Zhou, Ming Zhou, Zhaoyang Zeng, Xiaoling Li, Shourong Shen, Cijun Shuai, Guiyuan Li, Jiasheng Fang, Shuping Peng

**Affiliations:** 1 Cancer Research Institute, Central South University, Changsha, Hunan, P.R. China; 2 Cancer Research Institute, University of South China, Hengyang, Hunan, P.R. China; 3 Third Xiangya Hospital, Central South University, Changsha, Hunan, P.R. China; 4 State Key Laboratory of High Performance Complex Manufacturing, Central South University, Changsha, Hunan, P.R. China; 5 Department of Neurosurgery, Xiangya Hospital, Central South University, Changsha, Hunan, P.R. China; Vanderbilt University Medical Center, United States of America

## Abstract

**Background:**

MicroRNAs have been considered as a kind of potential novel biomarker for cancer detection due to their remarkable stability in the blood and the characteristics of their expression profile in many diseases.

**Methods:**

We performed microarray-based serum miRNA profiling on the serum of twenty nasopharyngeal carcinoma patients at diagnosis along with 20 non-cancerous individuals as controls. This was followed by a real-time quantitative Polymerase Chain Reaction (RT-qPCR) in a separate cohort of thirty patients with nasopharyngeal carcinoma and thirty age- matched non-cancerous volunteers. A model for diagnosis was established by a conversion of mathematical calculation formula which has been validated by analyzing 74 cases of patients with nasopharyngeal carcinoma and 57 cases of non-cancerous volunteers.

**Results:**

The profiles showed that 39 and 17 miRNAs are exclusively expressed in the serum of non-cancerous volunteers and of patients with nasopharyngeal carcinoma respectively. 4 miRNAs including miR-17, miR-20a, miR-29c, and miR-223 were found to be expressed differentially in the serum of NPC compared with that of non-cancerous control. Based on this, a diagnosis equation with Ct difference method has been established to distinguish NPC cases and non-cancerous controls and validated with high sensitivity and specificity.

**Conclusions:**

We demonstrate that the serum miRNA-based biomarker model become a novel tool for NPC detection. The circulating 4-miRNA-based method may provide a novel strategy for NPC diagnosis.

## Introduction

Nasopharyngeal carcinoma is rare in Europe, America and other western countries, while the rate of incidence is very high in China, Japan and other Southeast Asia [1], [2]. Nasopharyngeal disease is hard to be detected due to the hidden location and no obvious clinical manifestations in early stage. Nasopharyngeal carcinoma is often accompanied by early lymph node metastasis. The main clinical present screening method for nasopharyngeal carcinoma is to detect Epstein-Barr virus (EBV) infection in the serum, such as EBV-DNA, EBV-VCA-IgA and so on. But not all NPC carcinogenesis is associated with EBV, while EBV infection can be also observed in many other diseases (lymphoma, gastric cancer, breast cancer, pharyngitis, infectious mononucleosis psychosis, etc). Even healthy individuals could have EBV infection to a certain context. A large number of clinical experiments have showed that the sensitivity and specificity of diagnosis by using EBV-related detection for nasopharyngeal carcinoma screening is not high, which cannot meet the requirements as a diagnostic marker.

During the development and progression of the diseases, blood-related changes in the composition are often observed prior to morphological changes, reflecting the body’s natural status. In addition, blood samples are obtained easily from patients. Hematology testing is a minimally invasive, easy clinical implementation for population screening. Protein and peptides in the serum were well studied as biomarker. However, protein and peptide composition in serum is very complex, and relatively high abundance of albumin and globulin seriously dramatically interferes with protein and peptide marker screening. In addition, blood proteins and peptides are susceptible to physiological state, lifestyles and in vitro environment, which lead to unsatisfactory repeatability and reliability. Therefore, exploring a novel strategy for molecular diagnosis markers from the blood is promising and challengeable.

MicroRNAs (miRNAs) are a family of small non-coding RNA molecules of about 18–23 nucleotides, negatively regulating the expression of protein-coding genes at the post-transcriptional level. They specifically target the mRNA by inhibiting the translation or directing the target mRNA into degradation. It plays a key role in the expression of certain tissue-specific gene closely related to the development of the diseases. MiRNAs are even playing a similar role as oncogenes or tumor suppressor[3]–[8]. Several research groups found that the similar level of miRNA was present in human serum and plasma, because of its presence in the blood circulation, so called the circulating miRNAs [8]–[10]. These miRNAs are not readily affected by endogenous and exogenous RNases in the blood. They are not degraded for a long time at room temperature and remain stable after repeated freezing and thawing. Moreover, they can tolerate varieties of pH conditions. In addition, different healthy individuals have fairly consistent level of circulating miRNAs expression, no significant individual differences, but the conditions of different diseases can significantly affect the level of miRNAs in serum which has a good repeatability and comparability [11]. Considering circulating miRNAs as disease marker has the following advantages: 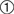  Related to the diseases closely, especially in tumors; 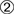  Blood samples are available non-invasively; 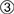 ; Subtle individual differences in the population; 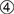 ; Stably present in serum/plasma; 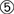  Mature methology of miRNA detection technology. These advantages indicate that circulating miRNAs molecular markers can be potential biomarkers for the diagnosis of nasopharyngeal carcinoma.

In this study, circulating miRNA expression profile was identified in the serum by comparing with the patients of nasopharyngeal carcinoma and non-cancerous control. Several potential serum miRNAs were validated as the diagnosis marker of nasopharyngeal carcinoma. A novel strategy for the diagnosis of NPC has been developed successfully. This may provide some references for the clinical diagnosis of NPC patients.

## Materials and Methods

### Patients and Serum Samples

Following ethical approval and written informed consent, serum samples were collected from 303 individuals, including 160 consecutive NPC patients and 143 age- and gender-matched non-cancerous donors who served as controls for this study. All samples were collected in consenting individuals from the First and Third Xiangya hospital affiliated by Central South University. The sample collection was performed according to the protocols approved by the ethics committee of the First Xiangya hospital and the Third Xiang Hospital. All the patients had been diagnosed as NPC pathologically and their relevant demographic and clinical pathological details were obtained (Table 1). The blood samples were collected prior to any therapeutic procedures, such as chemotherapy and radiotherapy. Control subjects were recruited from a large pool of individuals seeking a routine Healthy Physical Examination in the First Xiangya Hospital and Third Xiangya Hospital. The individuals with no evidence of cancer were selected as non-cancerous control subjects. Controls were matched to the patients based on age, gender and ethnicity.

**Table 1:** Demographic and clinical features of NPC patients and non-cancerous subjects.

Variable		NPC(n = 160)	Control(n = 143)	p-value(NPC vs. Control)
Average ages(years)				
**Age(years)**		46.41±10.74	46.85±12.92	0.746
**Sex**	**Male**	98	82	0.489
	**Female**	62	61	
**Histological types**		Squamous carcinoma		
**TNM stage**	I	2		
	II	25		
	III	55		
	IV	64		
	**Unknown**	14		

### RNA Isolation from Human Serum Samples

For the serum collection, venous blood (5 ml) was collected in the morning before breakfast from non-cancerous control and NPC patients before any therapeutic procedures including surgical resections of the primary tumors and put at room temperature for 1 h, centrifuged at 1,600 g for 10 min at 4°C. The serum was collected gently for store at −80°C or for the following step. For all experiments, 500 µl of human serum was used and total RNA was extracted and eluted in 60 µl of RNase-free water using a mirVana PARIS kit (#1556; Ambion) following the manufacturer’s protocol for liquid samples.

### MiRNA Profiling using TaqMan Low-Density Array

The sample for the profiling assay with TaqMan Low-Density Array is from the NPC serum pooled from 20 NPC or 20 non-cancerous volunteers. Technologically, miRNA expression in the serum from non-cancerous volunteers and NPC patients was profiled with TaqMan Human MiRNA Arrays, according to the manufacturer’s recommended protocol (Applied Biosystems.). This is a version of the TaqMan Low-Density Array (TLDA) quantitative reverse transcription polymerase chain reaction (qRT-PCR) profiling platform. Briefly, for microarray analysis, miRNAs was reverse transcribed using the TaqMan miRNA Reverse Transcription Kit. Three micro liter of human serum miRNA were added to each of the multiscribe reverse transcription reactions. 900 µl sample/master mix was loaded into Multiplex pool of the array, the array was then centrifuged and mechanically sealed with the Applied Biosystems sealer device. qRT-PCR was performed on an Applied BioSystems 7900HT thermocycler following the manufacturer’s recommended cycling conditions. Data were analyzed with SDS version 2.0 (Applied BioSystems). The average levels of 5 miRNAs which are commonly used as internal control of serum miRNA analysis. The differentially expression level of serum miRNA was calculated by the following equation:
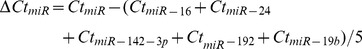
(1)


### Validation of Differentially Expressed miRNAs by qRT-PCR

Using spiked-in C. elegans miRNAs as internal reference controls, serum samples from 30 NPC patients and 30 non-cancerous volunteers respectively were used for the validation of differentially expressed miRNAs in serum. Given the early stage of plasma/serum miRNA study, no established endogenous small miRNAs were acted as the controls for normalization of technical variations in sample processing or potential variation in sample quality. Normalization by matching the amount of loaded RNA in the RT reaction is not a feasible approach because the RNA content in plasma or serum varies considerably with physical or pathological states. During the RNA extraction for qRT-PCR analysis, cel-miR-39 and cel-miR-238 was spiked at a fixed concentration as internal control. We therefore chose to use a fixed volume of RNA elute (5 µl) from a given volume of starting serum (500 µl), and normalized by the added exogenous cel-miR-39 and cel-miR-238.

MiScript PCR System kit series (miScript Reverse Transcription Kit, miScript SYBR Green PCR Kit, Qiagen) were used to quantify specific miRNAs. The fold change of differentially expressed miRNA level was calculated with 2^−ΔΔCt^.
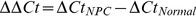
(2)

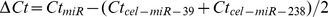
(3)


## Results

### Differentially Expressed Serum miRNA Profile in Nasopharyngeal Carcinoma from Non-cancerous Individuals Identified with TLDA miRNA Array

MiRNAs were extracted from the patients with nasopharyngeal carcinoma and non-cancerous controls. Paired serum samples were prepared by a mixture of equal volume of 20 serum samples from either NPC patients or non-cancerous population, the ratio of male/female in each group is 12∶8, the mean age in NPC group is 46.6 and 46.7 years in non-cancerous controls, respectively. There is no difference statistically between the two groups for age and sex (Table 2).

**Table 2:** Age and sex constitution of 20 NPC patients and 20 non-cancerous volunteers from whose serum collected used for TLDA miRNA array.

NPC		Non-cancerous controls	
Sex	Age	Sex	Age
M	40	M	62
F	31	M	38
M	43	F	55
M	47	M	38
F	52	M	46
F	29	F	49
M	36	F	52
M	49	F	50
M	51	M	22
M	66	F	52
M	47	M	46
F	53	F	42
M	61	F	56
M	51	M	52
M	24	F	37
M	54	M	42
F	55	M	32
F	53	M	61
F	45	M	57
F	44	M	45

The miRNA expression profiling of the serum in NPC patients or non-cancerous controls was analyzed. The results show that there are 86 miRNAs co-expressed in serum of NPC patients and non-cancerous volunteers,39 miRNAs (let-7c, let-7d, miR-106b, miR-10b, miR-125a, miR-132, miR-133a, miR-139-5p, miR-148b, miR-15b, miR-193a, miR-200b, miR-21, miR-224, miR-27a, miR-324-3p, miR-375, miR-423-5p, miR-494, miR-525-3p, miR-590-5p, miR-652, miR-660, miR-95, miR-10b*, miR-151-3p, miR-109b, miR-193b*, miR-223*, miR-30b*, miR-550*, miR-564, miR-584, miR-601, miR-632, miR-643, miR-661, miR-92a-2*, miR-942) are exclusively expressed in non-cancerous serum, and 17 miRNAs (RNU44, miR-100, miR-124, miR-130a, miR-143, miR-148a, miR-215, miR-28-3p, miR-28-5p, miR-323-3p, miR-328, miR-429, miR-144*, miR-30a*, miR-335*, miR-425*, miR-565) are exclusively expressed in serum of patients with nasopharyngeal carcinoma (Fig. S1, Table S1,2). Although 56 miRNAs differentially expressed in nasopharyngeal carcinoma and non-cancerous controls, however, the vast majority of these miRNAs expression is very low (Ct value is more than 35), a reliable range of Ct values should be less than 35 for the chip analysis. Therefore, we selected differentially expressed miRNAs among those either in NPC or non-cancerous controls whose Ct is less than 35. Currently, there is no accepted reliable reference gene (housekeeping gene) in serum as internal control. The combination of miRNAs used in other literatures [9], [12], [13] was used as internal control, including miR-16, miR-24, miR-142-3p, miR-19b, miR-192. Comparative analysis of microarray data was performed with Equation (1).

The principles for selecting differentially expressed serum miRNA are: (1) The serum levels of miRNA Ct value in each group is less than 35; (2) The fold change of miRNAs in serum between NPC patients and non-cancerous controls is more than 2, suggesting the fold change of expression level is more than 4 times; (3) The miRNAs reported by other literatures as differentially expressed miRNAs between NPC and non-cancerous controls are optimal; (4) Those miRNAs reported with clinical diagnosis value in the serum of patients with other tumors were of priority. Based on the above principles, the initial selection of 18 serum miRNAs were subjected to the next step verification: miR-148a, miR-151-3p, miR-20a, miR-29c, miR-30c, miR-323-3p, miR-130a, miR-17, miR-222, miR-486-3p, miR-21, miR-106b, miR-223, miR-155, miR-106a, miR-145, miR-143, miR-92a.

### Validation of the miRNAs Differentially Expressed in NPC Patients and Non-cancerous Controls with qRT-PCR

**Figure 1:**
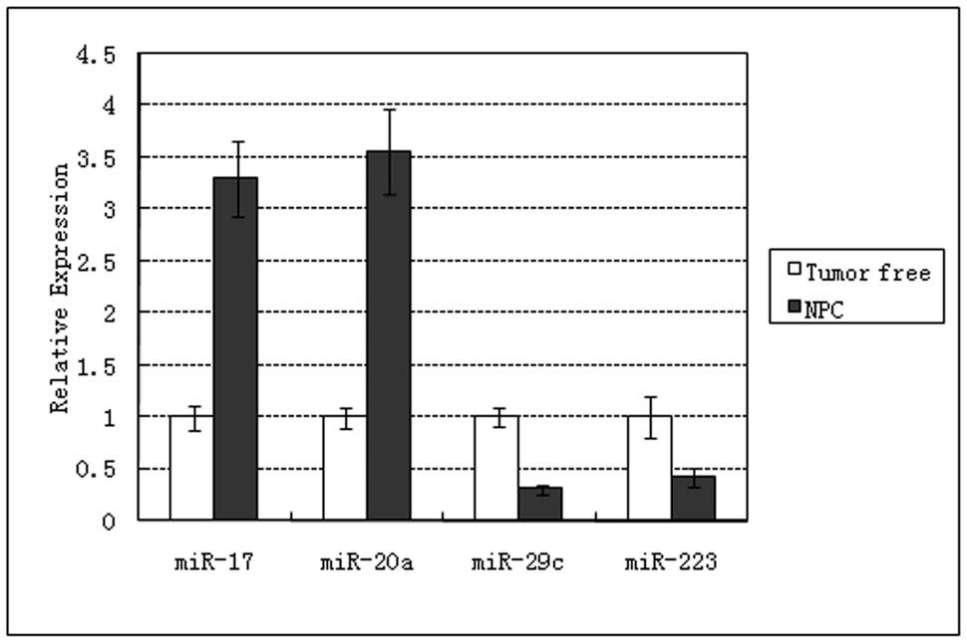
Expression of miR-17, miR-20a, miR-29c and miR-223 selected with miRNA array by a real-time quantitative Polymerase Chain Reaction (RT-qPCR), showing differentially expressed between NPC and non-cancerous group.

As the number of the miRNAs first screened with MiRNA array is too much, which are not suitable for the validation of one by one, we first mixed the serum for second screening. Each set includes six serum pooled either from non-cancerous volunteers or NPC patients and matched in pairs (the ratio of male/female in each pair is 1∶1, the average age difference is less than 3 years). We used six groups of pooled serum (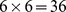
cases) from non-cancerous controls and pooled serum (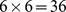 patients) from nasopharyngeal carcinoma patients for secondary screening. Serum RNA extraction process by adding cel-miR-39 and cel-miR-238 as a reference (two experiments confirmed that there is no expression of cel-miR-39 and cel-miR-238 in human serum (Fig. S2). Quantitative PCR was performed to screen miRNAs differentially expressed between two groups. Data were analyzed using Equation (2) and (3). Two elevated miRNAs (miR-17, miR-20a) and two down-regulated miRNAs (miR-29c, miR-223) in NPC serum were identified (Fig. 1).

**Figure 2:**
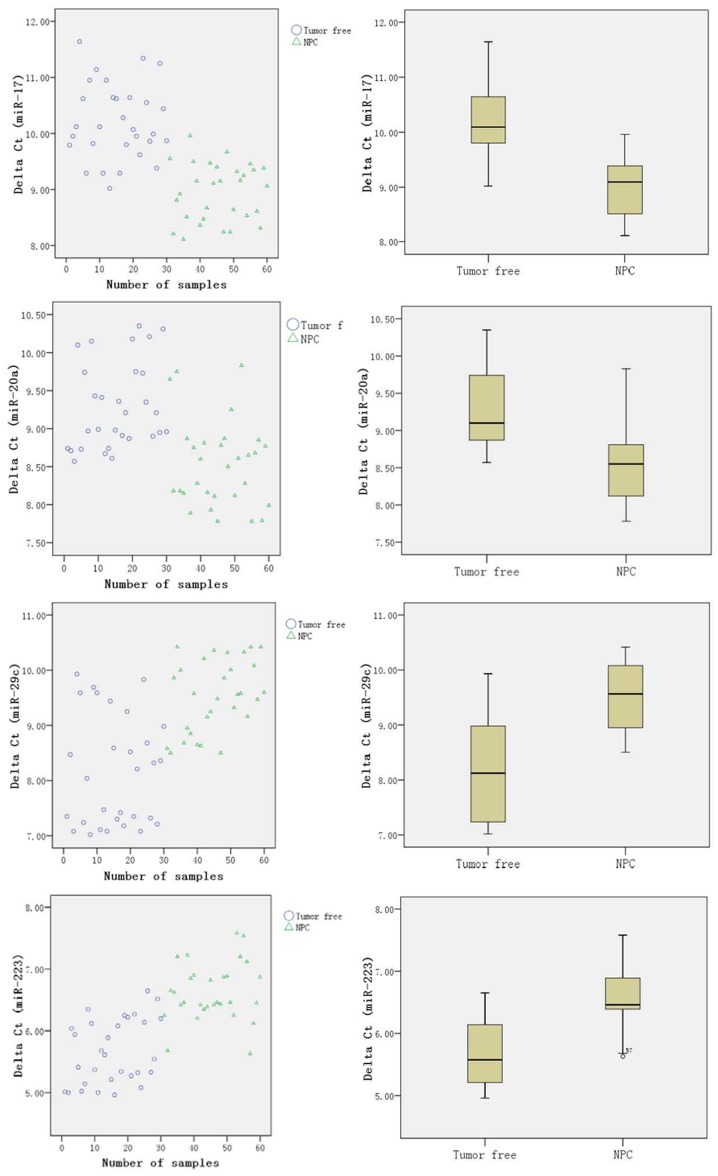
Expression distribution of miR-17, miR-20a, miR-29c and miR-223 in 60 serum samples (Left: scatter; Right: Box) by RT-qPCR. The levels of these miRNAs are a bit overlapping between NPC patients and non-cancerous controls though they are differentially expressed.

The four miRNAs differentially expressed in the serum of NPC patients compared with those in non-cancerous controls were selected for the validation for quantitative PCR detection. Exogenous cel-miR-39 and cel-miR-238 were added as an internal reference. Data were analyzed with Equation (2) and Equation (3). The expression level of four miRNAs in 60 serum samples (including 30 NPC patients and 30 non-cancerous volunteers) were analyzed (Fig. 2).

### Prediction of NPC with 4-miRNA- Based Diagnosis Model

We are exploring for nasopharyngeal carcinoma serum differentially expressed miRNAs, which are expected to be used in clinical diagnosis of NPC. As the difference of the selected four miRNAs between in NPC patients and the non-cancerous is not “positive" or “negative" in expression, any of them is not suitable to be used to diagnose NPC by a single factor. Stable and reliable reference control or absolute quantification is required for the diagnosis with miRNAs in serum. However, there is no reliable internal reference of miRNA in serum currently, and absolute quantification is difficult. It is not convenient for clinical application of serum miRNAs biomarkers. According to the actual screening of miRNA expression characteristics, we creatively designed “Ct difference method," as the diagnosis model of NPC.

Assuming that miR-a is down-regulated and miR-b is up-regulated in NPC, 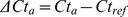;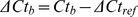.

Here Ct of miR-a is represented by Ct_a_; Ct of miR-b is indicated with Ct_b_,a diagnosis indicators of NPC with serum miRNA may be:
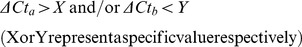
(4)


As there is no reliable serum miRNA internal reference generally accepted, ΔCt_a_ cannot be obtained. An idea is that the above diagnosis of the mathematical formula can be converted into.
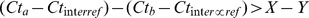
(5)as Ct_a_ and Ct_b_ are from the same batch results for the same sample, therefore Ct_inter ref_ is same.



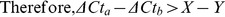
(6)Based on the above mathematical formula, the impact of internal reference may be eliminated. It is not required in the diagnosis formula any more. In order to set up a diagnosis standard, it is necessary to set reference values assumed to be A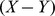.

**Figure 3:**
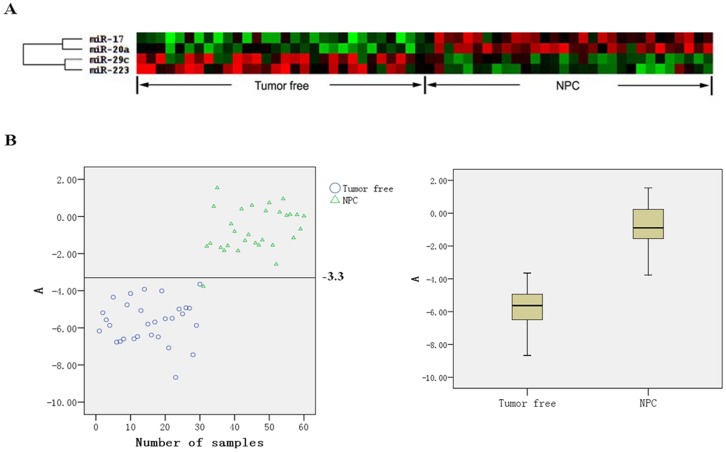
A: miR-17, miR-20a, miR-29c and miR-223 cluster analysis by Cluster 3.0 in 60 cases serum of NPC patients and non-cancerous control; B: A value distribution in 60 serum sample (Left: scatter; Right: Box). This shows that A value ( = 3.3) could discriminate the NPC patients and non-cancerous control well.



Therefore, the diagnosis method should have one or more miRNAs involved, and should include the up-regulated and down-regulated miRNAs. Theoretically, multiple miRNAs combined diagnosis can increase diagnostic reliability. According to the difference and stability of these four screened miRNAs in each serum sample, miR-17, miR-20a up-regulated and miR-29c, miR-223 down-regulated in NPC patients were selected as the serological diagnosis indicators for nasopharyngeal carcinoma. The four miRNAs were clustered well between the NPC patients and non-cancerous controls analyzed by Cluster 3.0 (Fig. 3A). According to the experimental data, we calculated the value of 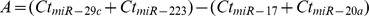. A value was obtained with the RT-qPCR of 60 cases serum (Fig. 3B, left). We take two sets of data closest to the 10% of the data (non-cancer group: −4.01, −3.92, −3.65; nasopharyngeal carcinoma group: −3.77, −2.58, −1.85), and calculated the mean value is −3.30 as reference value of diagnosis for nasopharyngeal carcinoma with serum miRNA, the establishment of the following diagnostic model (diagnostic equation):

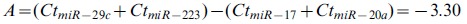



If the value of A is more than −3.30, the patient can be diagnosed as NPC; otherwise, A <−3.30 suggesting non-nasopharyngeal carcinoma. The diagnostic sensitivity was 96.7% (29/30), specificity was 100% (30/30), only 1 false negative (Fig. 3B).

In order to validate the diagnosis model, other separate panel serum samples (including 57 cases serum from non-cancerous population and 74 cases serum of NPC patients were used. The diagnostic sensitivity was 97.3% (72/74), specificity rate was 96.5% (55/57) (Table 3).

**Table 3:** Validation of the serum 4-miRNA based diagnosis model in 131 cases of serum samples.

Group	Correctly Classified	Healthy	NPC
Non-cancerous Controls	96.50%	55	2
NPC	97.30%	72	2

### Correlation of Serum miRNA Expression with Clinical Prognosis

**Figure 4:**
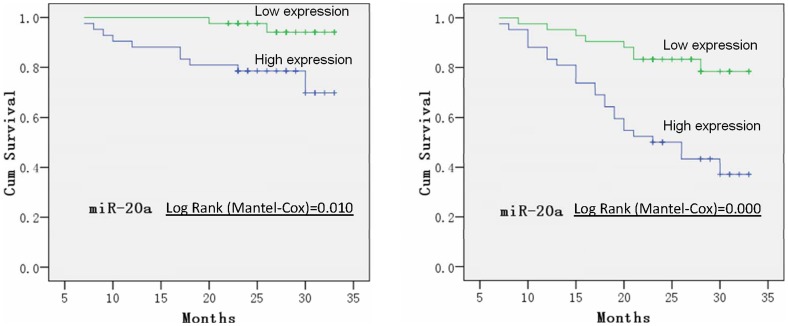
Kaplan-Meier survival curve analysis of miR-20a (Left: total survival curve; right: disease-free survival curve). Log Rank value = 0.010 and 0.000, respectively, analyzed by Software SPSS.

To understand the NPC-associated serum miRNA expression and clinical prognosis, we followed up 84 patients by serum miRNA detection of miR-17, miR-20a, miR-29c, and miR-223. The association between miRNA expression of the patients and disease-free survival were analyzed with Kaplan-Meier survival curve with SPSS software. It showed that the expression level of miR-20a is negatively associated with the prognosis of nasopharyngeal carcinoma. The higher expression of miR-20a in serum is, the worse outcome for the NPC patient would be. The overall survival curve and disease-free survival curves have demonstrated this point (Fig. 4). In all 32 cases of recurrence and death, 24 cases (including 9 deaths) have miR-20a expression and only 8 cases (2 deaths) have low miR-20a expression. The Log Rank value is 0.010 and 0.000 for miR-20a associated with overall survival and disease-free survival respectively, which indicates the poor prognosis of the patients with high expression level of miR-20a than the ones with low level of miR-20a statistically.

## Discussions

The emergence of miRNAs as modulators of gene expression and their established role in carcinogenesis identifies them as obvious candidate diagnostic and prognostic indicators for malignancy [14]. In nasopharyngeal carcinoma, host encoded miRNAs and Epstein-Barr virus encoded miRNAs play key roles in almost all the steps of epithelia cell carcinogenesis, including epithelial-mesenchymal to stem-like transition, cell growth, migration, invasion, and tumorigenesis[15]–[20].

The recent surge of reports documenting altered miRNAs in the circulation of cancer patients has given momentum to their putative role as noninvasive cancer biomarkers. The prognostic value of these miRNAs in the circulation has been shown for non-small-cell lung cancer [21], [22], breast cancer [23], head and neck squamous cell carcinoma [24], malignant astrocytomas [25],etc. The importance of circulating miRNA in the prognostic value in nasopharyngeal carcinoma has been gained much attention. Recently, Wong A.M. group reported that 12 up-regulated EBV microRNAs (BART1-3p, 2–5p, 5, 6–5p, 6-3p, 7, 8, 9, 14, 17-5p, 18-5p, 19-3p) in paired NPC biopsies and these microRNAs are distinct present in the serum of NPC patients [26]. They indicated that EBV microRNAs in serum of NPC patients are generally more up-regulated than those of human origin. Further investigation of potential EBV microRNA target genes revealed inhibition of tumor suppressor genes (eg, PTEN) and extensive deregulation of several pathways frequently involved in NPC (eg, Wnt signaling). Claire Gourzones group demonstrated that the BART miRNAs encoded by EBV are released in the extra-cellular space by NPC cells being associated with secreted exosomes at least to a large extent. These miRNAs were detected with a good selectivity in plasma samples from NPC xenografted nude mice as well as NPC patients [27]. These studies provide good basis of the role of circulating miRNAs encoded by EBV genome.

In this study, four miRNAs from human origin in the serum were selected as the diagnosis biomarkers of nasopharyngeal carcinoma. The detection of 4 miRNA levels in the biopsy tissue of NPC patients at different stages are also performed (Fig. S3 [28]) (GSE32906). MiR-17 and miR-20a are the members of miR-17 family, which includes miR-17-5p, miR-20a/b, miR-106a/b and miR-93. The family has a double-sided role in tumors. Several groups reported that miR-17 and miR-20a in colon cancer is highly expressed in plasma and tissues [29], [30]. They act as oncogenes in the lymphoma and vascular tumor [31], and tumor suppressor in breast cancer [32], [33]. Up-regulated mir17 in nasopharyngeal carcinoma biopsies was also reported by Chen HC group [15]. Hui et al [34]found that miR-20a is highly expressed in cancer tissue by comparing the miRNA profiles of head and neck squamous cell carcinoma and normal tissue. However, to the best of our knowledge, the association of miR-20a with NPC was not reported. Our results showed that the expression of MiR-17 and miR-20a was not only elevated in the serum but also up-regulated in the different stages of NPC progression and even higher level in metastasis, functioning as oncogenic miRNAs.

MiR-29c is a member of the miR-29 family as well as miR-29a and miR-29b. There is subtle differences only in the 3′ end nucleotides among the three members. In many tumors, miR-29c expression is decreased to different extent, which regulates MCL-1, TCL-1 and other cancer-related genes and other genes involved in tumor onset and progression[35]-[37]. In addition, miR-29 family members (miR-29a, miR-29b, miR-29c) can inhibit p85 alpha (PI3-kinase subunit), CDC42 and promote p53 expression, then induce cell apoptosis. Therefore, reduced miR-29 expression leads to reduced p53 expression [38] and the reduction of apoptosis, and promotes the carcinogenesis. Reduced miR-29c up-regulate the expression of extracellular matrix proteins, thereby enhancing cell invasion and migration, promoting tumor cell metastasis. More than one literature reported that miR-29c is reduced in nasopharyngeal carcinoma [15], [18]. We also found that the reduced miR-29c expression in the progression of NPC and more reduced in the biopsies with metastasis by laser micro dissection (Fig. S3).

The role of miR-223 is still controversial. It can promote the production of normal neutrophils cells which is well studied in the lymphatic system disorders [5], [39]–[42]. It is a potential marker for ovarian cancer recurrence [43]. Increased miR-223 expression in the serum/plasma was revealed in lung cancer [44], liver [45], diabetes [46] and sepsis [47].However, miR-223 in esophageal cancer was identified to directly target tumor metastasis-related genes [48]. In consistent with this notion, our results further confirm that miR-223 expression is reduced in serum of nasopharyngeal carcinoma patients, which is consistent to be a tumor suppressor gene. However, the expression of miR-223 is not altered so much compared with normal nasopharyngeal epithelial (Fig. S3). That is, on one hand, the changes of the some miRNAs level in serum are associated with the pattern in the primary tissue, on the other hand, the levels of other miRNAs are somehow independent from tissue specimens, indicating the serum miRNAs have different sources and secretary mechanism [21], [22], [24], [49].

Although some studies screening miRNAs differentially, it is still difficult to develop diagnostic indicators as there is no generally accepted reliable internal control. This has been a bottleneck for direct clinical application. In this study, we proposed the “Ct difference method" to avoid the requirement of internal reference. It should be more feasible to develop circulating miRNA as diagnostic biomarker as neither absolute quantification of serum miRNA nor internal reference is required. We established a mathematical formula based on selected miRNAs for the detection of NPC with QIAGEN’s miScript PCR System kit series. Though different miRNAs reverse transcription and real-time PCR kit may lead to different Ct, as long as the relevant ΔCt values keep the same, the difference will not affect our diagnostic results. Therefore, we may not conclude that the “A" value will keep the same with the reagents from different manufacturers. It can be adjusted to an optimum. In a summary, the diagnosis formula may be used for the judgment for the diagnosis of NPC and the “Ct difference method" proposed in this study provides a new strategy using serum miRNA biomarker for the detection of NPC.

## Supporting Information

Figure S1 **Constitution of miRNA profile in non-cancerous control and nasopharyngeal carcinoma patients.**
(TIF)

Figure S2 **No detectable expression of cel-miR-39 and cel-miR-238 in human serum with quantitative PCR, while hsa-miR-16 is expressed in human serum.**
(TIF)

Figure S3 **Dynamic miRNA expression of hsa-miR-17, hsa-miR-20a, hsa-miR-29c, hsa-miR-223 in NPC biopsies at different clinical stages.** The microdissection was performed with Methyl Green staining to separate tumor cells to non-tumor cells. Total RNA was extracted using Trizol® reagent (Invitrogen) from samples. The Ambion Illumina Total RNA Amplification Kit was used to synthesize biotinylated cDNA. MicroRNA expression profiling kit contains primers for 1146 human miRNAs. The biotinylated cDNAs were hybridized with microRNA-specific oligonucleotides. Polymerase Chain Reactions (PCR) were performed with fluorescently labelled universal primers, followed by hybridizing of the fluorescently labelled, single-stranded PCR products to capture beads. The fluorescent signals were then detected by Illumina’s iScan System.
(TIF)

Table S1 **Serum miRNA profile of non-cancerous volunteers with Taqman Human MiRNA array (detectable miRNA listed) including plate A and B.**
(DOC)

Table S2 **Serum miRNA profile of nasopharyngeal carcinoma patients with Taqman Human MiRNA array (detectable miRNA listed): plate A and B.**
(DOC)
