# Supplementary figures and images for: Circulating miR-17, miR-20a, miR-29c, and miR-223 Combined as Non-Invasive Biomarkers in Nasopharyngeal Carcinoma
Source: PLoS One. 2012 Oct 8;7(10):e46367. doi: 10.1371/journal.pone.0046367 (PMC3466268; doi:10.1371/journal.pone.0046367)

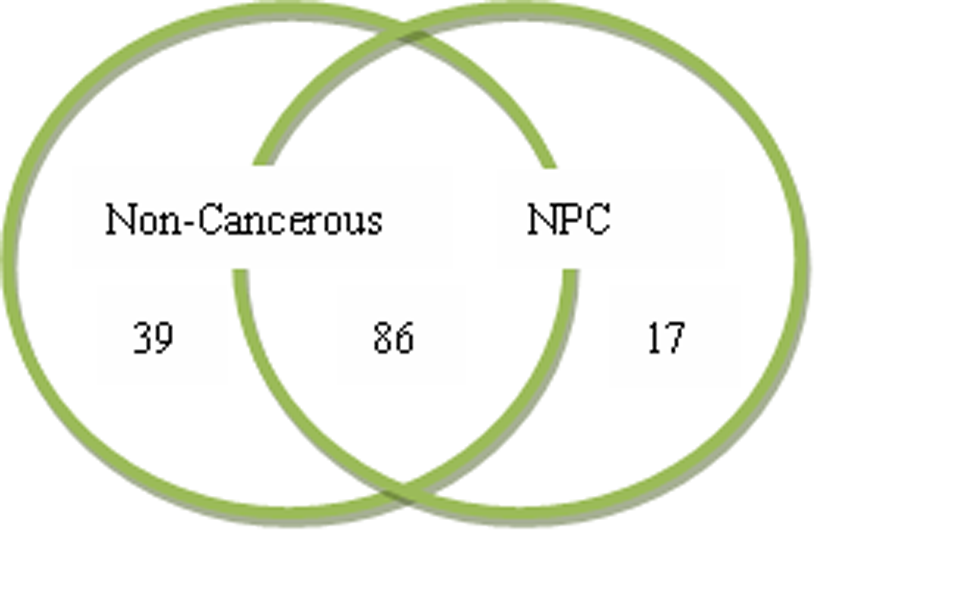

Supplement: Figure S1 — Constitution of miRNA profile in non-cancerous control and nasopharyngeal carcinoma patients. (TIF) [file pone.0046367.s001.tif]

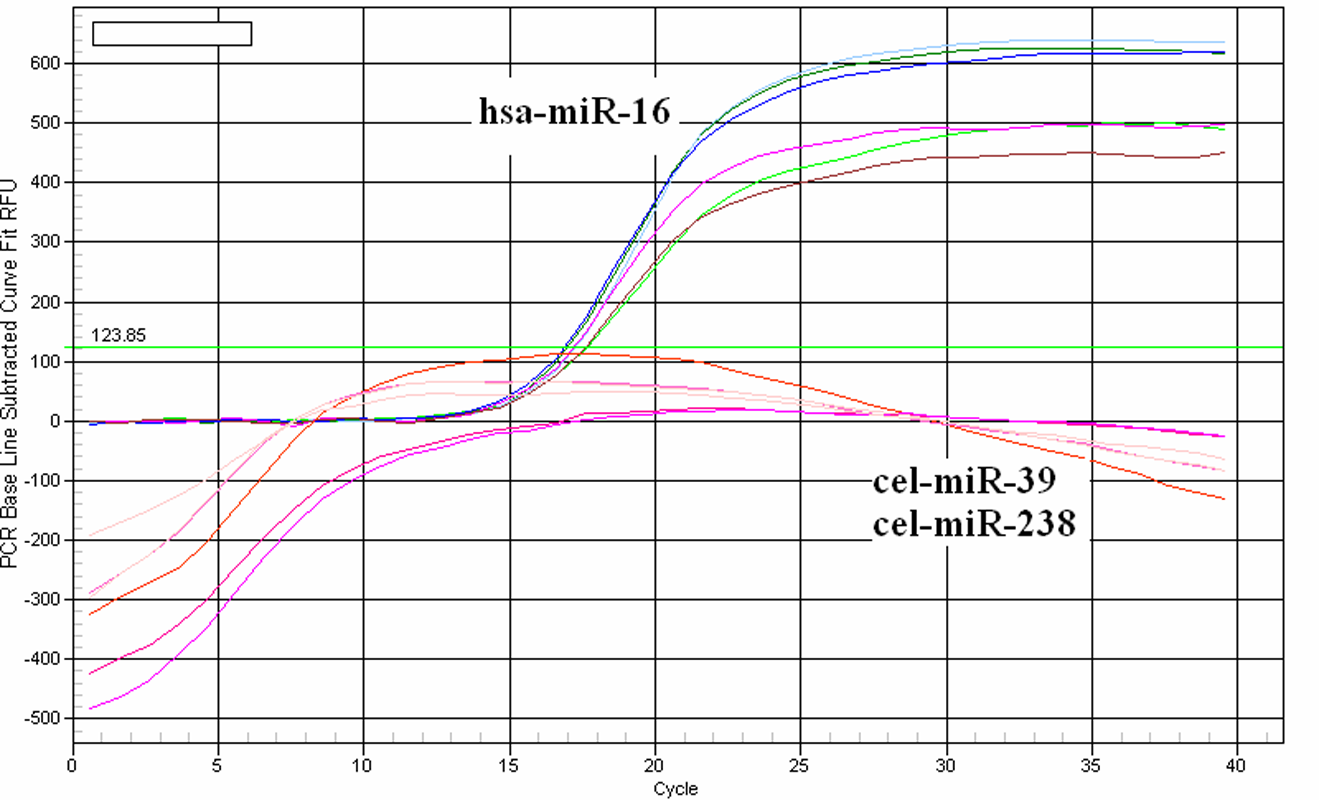

Supplement: Figure S2 — No detectable expression of cel-miR-39 and cel-miR-238 in human serum with quantitative PCR, while hsa-miR-16 is expressed in human serum. (TIF) [file pone.0046367.s002.tif]

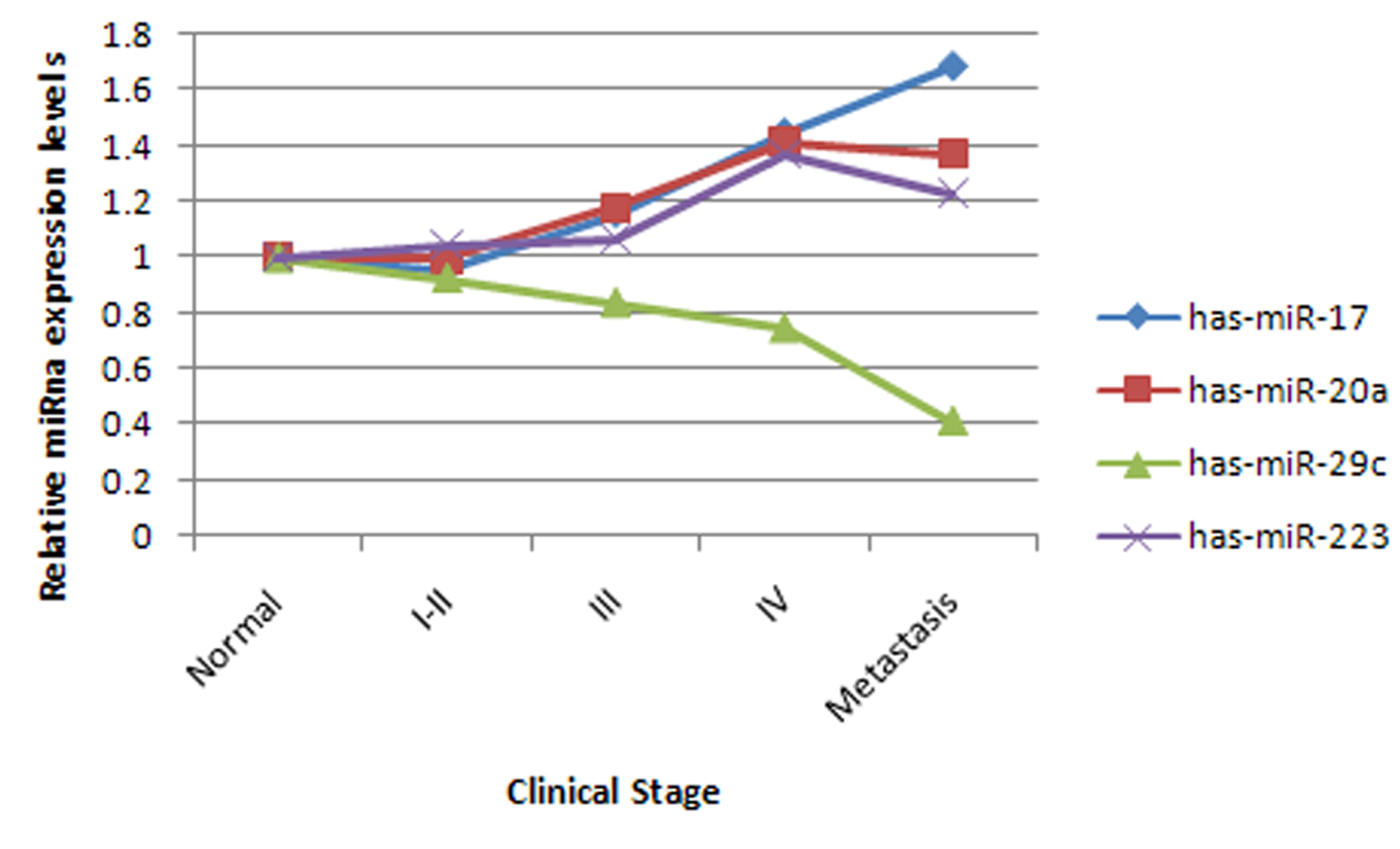

Supplement: Figure S3 — Dynamic miRNA expression of hsa-miR-17, hsa-miR-20a, hsa-miR-29c, hsa-miR-223 in NPC biopsies at different clinical stages. The microdissection was performed with Methyl Green staining to separate tumor cells to non-tumor cells. Total RNA was extracted using Trizol® reagent (Invitrogen) from samples. The Ambion Illumina Total RNA Amplification Kit was used to synthesize biotinylated cDNA. MicroRNA expression profiling kit contains primers for 1146 human miRNAs. The biotinylated cDNAs were hybridized with microRNA-specific oligonucleotides. Polymerase Chain Reactions (PCR) were performed with fluorescently labelled universal primers, followed by hybridizing of the fluorescently labelled, single-stranded PCR products to capture beads. The fluorescent signals were then detected by Illumina’s iScan System. (TIF) [file pone.0046367.s003.tif]
