# Supplementary material for: Circulating miR-17, miR-20a, miR-29c, and miR-223 Combined as Non-Invasive Biomarkers in Nasopharyngeal Carcinoma
Source: PLoS One. 2012 Oct 8;7(10):e46367. doi: 10.1371/journal.pone.0046367 (PMC3466268; doi:10.1371/journal.pone.0046367)
Supplement: Table S1 — Serum miRNA profile of non-cancerous volunteers with Taqman Human MiRNA array (detectable miRNA listed) including plate A and B. (DOC) [file pone.0046367.s004.doc]

**Table S1 Serum miRNA profile of non-cancerous volunteers with Taqman Human MiRNA array (detectable miRNA listed) plate A**

| miRNA | Avg Ct |
| --- | --- |
| hsa-miR-223-4395406 | 23.733 |
| hsa-miR-486-5p-4378096 | 24.677 |
| hsa-miR-16-4373121 | 25.287 |
| MammU6-4395470 | 26.23 |
| hsa-miR-484-4381032 | 26.967 |
| hsa-miR-191-4395410 | 27.171 |
| hsa-miR-320-4395388 | 27.701 |
| hsa-miR-106a-4395280 | 28.443 |
| hsa-miR-19b-4373098 | 28.446 |
| hsa-miR-126-4395339 | 28.561 |
| hsa-miR-222-4395387 | 28.653 |
| hsa-miR-146a-4373132 | 28.725 |
| hsa-miR-24-4373072 | 28.993 |
| hsa-miR-150-4373127 | 29.589 |
| hsa-miR-17-4395419 | 30.032 |
| hsa-miR-92a-4395169 | 30.197 |
| hsa-miR-197-4373102 | 30.283 |
| hsa-miR-186-4395396 | 30.289 |
| hsa-miR-19a-4373099 | 30.944 |
| hsa-miR-342-3p-4395371 | 30.976 |
| hsa-miR-93-4373302 | 31.072 |
| hsa-miR-451-4373360 | 31.44 |
| hsa-miR-574-3p-4395460 | 31.614 |
| hsa-let-7b-4395446 | 31.802 |
| hsa-miR-26a-4395166 | 31.828 |
| hsa-miR-483-5p-4395449 | 32.164 |
| hsa-miR-885-5p-4395407 | 32.183 |
| hsa-miR-106b-4373155 | 32.411 |
| hsa-miR-25-4373071 | 32.544 |
| hsa-miR-29a-4395223 | 32.72 |
| hsa-miR-140-5p-4373374 | 32.745 |
| hsa-miR-146b-5p-4373178 | 32.764 |
| hsa-miR-425-4380926 | 32.844 |
| hsa-let-7e-4395517 | 32.88 |
| hsa-miR-142-3p-4373136 | 32.929 |
| hsa-miR-122-4395356 | 32.944 |
| hsa-miR-345-4395297 | 33.083 |
| hsa-miR-140-3p-4395345 | 33.101 |
| hsa-miR-374b-4381045 | 33.14 |
| hsa-miR-375-4373027 | 33.147 |
| hsa-miR-192-4373108 | 33.158 |
| hsa-miR-454-4395434 | 33.382 |
| hsa-miR-21-4373090 | 33.408 |
| hsa-miR-590-5p-4395176 | 33.464 |
| hsa-miR-26b-4395167 | 33.653 |
| hsa-miR-125a-5p-4395309 | 33.702 |
| hsa-miR-132-4373143 | 33.72 |
| hsa-miR-20a-4373286 | 33.726 |
| hsa-miR-193b-4395478 | 33.803 |
| hsa-miR-199a-3p-4395415 | 33.92 |
| hsa-miR-195-4373105 | 33.94 |
| hsa-miR-331-3p-4373046 | 33.946 |
| hsa-miR-494-4395476 | 34.023 |
| hsa-miR-30b-4373290 | 34.036 |
| hsa-let-7g-4395393 | 34.054 |
| hsa-miR-660-4380925 | 34.083 |
| hsa-miR-374a-4373028 | 34.137 |
| hsa-miR-95-4373011 | 34.48 |
| hsa-miR-133a-4395357 | 34.6 |
| hsa-miR-29c-4395171 | 35.181 |
| hsa-miR-27a-4373287 | 35.201 |
| hsa-miR-10b-4395329 | 35.296 |
| hsa-miR-20b-4373263 | 35.326 |
| hsa-miR-148b-4373129 | 35.356 |
| hsa-miR-30c-4373060 | 35.378 |
| hsa-miR-422a-4395408 | 35.501 |
| hsa-miR-139-5p-4395400 | 35.54 |
| hsa-miR-224-4395210 | 35.775 |
| hsa-let-7c-4373167 | 35.886 |
| hsa-miR-324-3p-4395272 | 36.165 |
| hsa-miR-103-4373158 | 36.209 |
| hsa-miR-340-4395369 | 36.305 |
| hsa-let-7d-4395394 | 36.368 |
| hsa-miR-486-3p-4395204 | 36.441 |
| hsa-miR-339-3p-4395295 | 36.685 |
| hsa-miR-145-4395389 | 36.818 |
| hsa-miR-485-3p-4378095 | 36.945 |
| hsa-miR-423-5p-4395451 | 37.018 |
| hsa-miR-365-4373194 | 37.387 |
| hsa-miR-200b-4395362 | 37.55 |
| RNU48-4373383 | 37.834 |
| hsa-miR-193a-5p-4395392 | 37.956 |
| hsa-miR-525-5p-4378088 | 38.062 |
| hsa-miR-15b-4373122 | 38.082 |
| hsa-miR-652-4395463 | 38.35 |

**plate B**

| miRNA | Avg Ct |
| --- | --- |
| hsa-miR-923-4395264 | 25.925 |
| MammU6-4395470 | 26.145 |
| hsa-miR-135a*-4395343 | 26.599 |
| hsa-miR-188-5p-4395431 | 30.267 |
| hsa-miR-93*-4395250 | 30.727 |
| hsa-miR-30e-4395334 | 30.912 |
| hsa-miR-766-4395177 | 30.985 |
| hsa-miR-126*-4373269 | 31.489 |
| hsa-miR-877-4395402 | 31.563 |
| hsa-miR-151-3p-4395365 | 31.687 |
| hsa-miR-30a-4373061 | 31.763 |
| hsa-miR-625*-4395543 | 31.949 |
| hsa-miR-801-4395183 | 32.22 |
| hsa-miR-378-4395354 | 32.321 |
| hsa-miR-30d-4373059 | 32.688 |
| hsa-miR-223*-4395209 | 33.629 |
| hsa-miR-509-3p-4395347 | 33.781 |
| hsa-miR-630-4380970 | 33.996 |
| hsa-miR-193b*-4395477 | 34.066 |
| hsa-miR-30e*-4373057 | 34.12 |
| hsa-miR-550*-4380954 | 34.526 |
| hsa-miR-650-4381006 | 34.735 |
| RNU48-4373383 | 35.235 |
| hsa-miR-760-4395439 | 35.376 |
| hsa-miR-942-4395298 | 35.467 |
| hsa-miR-661-4381009 | 35.682 |
| hsa-miR-768-3p-4395188 | 35.831 |
| hsa-miR-571-4381016 | 36.032 |
| hsa-miR-409-3p-4395443 | 36.047 |
| hsa-miR-632-4380977 | 37.308 |
| hsa-miR-92a-2*-4395249 | 37.875 |
| hsa-miR-564-4380941 | 38.095 |
| hsa-miR-99b*-4395307 | 38.353 |
| hsa-miR-601-4380965 | 38.624 |
| hsa-miR-30b*-4395240 | 39.255 |
| hsa-miR-623-4386740 | 39.348 |
| hsa-miR-643-4380997 | 39.429 |
| hsa-miR-584-4381026 | 39.597 |
| hsa-miR-190b-4395374 | 39.874 |
| hsa-miR-10b*-4395426 | 39.923 |
