# Supplementary material for: Circulating miR-17, miR-20a, miR-29c, and miR-223 Combined as Non-Invasive Biomarkers in Nasopharyngeal Carcinoma
Source: PLoS One. 2012 Oct 8;7(10):e46367. doi: 10.1371/journal.pone.0046367 (PMC3466268; doi:10.1371/journal.pone.0046367)
Supplement: Table S2 — Serum miRNA profile of nasopharyngeal carcinoma patients with Taqman Human MiRNA array (detectable miRNA listed): plate A and B. (DOC) [file pone.0046367.s005.doc]

**Table S2 Serum miRNA profile of nasopharyngeal carcinoma patients with Taqman Human MiRNA array (detectable miRNA listed) plate A**

| miRNA | Avg Ct |
| --- | --- |
| MammU6-4395470 | 24.742 |
| hsa-miR-223-4395406 | 25.392 |
| hsa-miR-16-4373121 | 26.144 |
| hsa-miR-486-5p-4378096 | 26.533 |
| hsa-miR-17-4395419 | 28.77 |
| hsa-miR-191-4395410 | 29.135 |
| hsa-miR-484-4381032 | 29.629 |
| hsa-miR-106a-4395280 | 29.641 |
| hsa-miR-320-4395388 | 29.693 |
| hsa-miR-19b-4373098 | 29.749 |
| hsa-miR-126-4395339 | 29.935 |
| hsa-miR-24-4373072 | 30.447 |
| hsa-miR-146a-4373132 | 30.916 |
| hsa-miR-20a-4373286 | 31.223 |
| hsa-miR-222-4395387 | 31.52 |
| hsa-miR-186-4395396 | 31.535 |
| hsa-miR-486-3p-4395204 | 31.556 |
| hsa-miR-92a-4395169 | 31.573 |
| hsa-miR-451-4373360 | 31.579 |
| hsa-miR-885-5p-4395407 | 31.859 |
| hsa-miR-19a-4373099 | 32.083 |
| hsa-miR-150-4373127 | 32.09 |
| hsa-miR-122-4395356 | 32.258 |
| RNU48-4373383 | 32.424 |
| hsa-let-7b-4395446 | 32.675 |
| hsa-miR-574-3p-4395460 | 32.808 |
| hsa-miR-30c-4373060 | 32.845 |
| hsa-miR-197-4373102 | 32.849 |
| hsa-miR-342-3p-4395371 | 33.266 |
| hsa-miR-140-5p-4373374 | 33.437 |
| hsa-miR-93-4373302 | 33.528 |
| hsa-miR-192-4373108 | 33.565 |
| hsa-miR-142-3p-4373136 | 33.633 |
| hsa-miR-26b-4395167 | 33.727 |
| hsa-miR-374b-4381045 | 33.897 |
| hsa-miR-454-4395434 | 33.974 |
| hsa-let-7e-4395517 | 33.986 |
| hsa-miR-25-4373071 | 34.037 |
| hsa-miR-26a-4395166 | 34.212 |
| hsa-miR-30b-4373290 | 34.331 |
| hsa-miR-28-3p-4395557 | 34.454 |
| hsa-miR-199a-3p-4395415 | 34.486 |
| hsa-miR-483-5p-4395449 | 34.618 |

| hsa-miR-345-4395297 | 34.653 |
| --- | --- |
| hsa-miR-145-4395389 | 34.711 |
| hsa-miR-195-4373105 | 34.724 |
| hsa-miR-148a-4373130 | 34.745 |
| hsa-miR-29a-4395223 | 34.8 |
| hsa-miR-331-3p-4373046 | 34.881 |
| hsa-miR-339-3p-4395295 | 34.885 |
| hsa-miR-193b-4395478 | 34.906 |
| hsa-miR-485-3p-4378095 | 34.957 |
| hsa-miR-340-4395369 | 35.059 |
| hsa-miR-143-4395360 | 35.119 |
| hsa-miR-20b-4373263 | 35.196 |
| hsa-miR-146b-5p-4373178 | 35.429 |
| hsa-miR-28-5p-4373067 | 35.461 |
| hsa-miR-100-4373160 | 35.497 |
| hsa-miR-323-3p-4395338 | 35.527 |
| hsa-miR-140-3p-4395345 | 35.533 |
| hsa-miR-103-4373158 | 35.607 |
| hsa-miR-215-4373084 | 35.615 |
| hsa-let-7g-4395393 | 35.957 |
| hsa-miR-374a-4373028 | 36.116 |
| hsa-miR-422a-4395408 | 36.432 |
| hsa-miR-425-4380926 | 36.447 |
| hsa-miR-130a-4373145 | 36.467 |
| RNU44-4373384 | 36.743 |
| hsa-miR-328-4373049 | 36.775 |
| hsa-miR-365-4373194 | 37.483 |
| hsa-miR-429-4373203 | 38.188 |
| hsa-miR-29c-4395171 | 38.31 |
| hsa-miR-124-4373295 | 39.215 |

**Plate B**

|  | miRNA | | Avg Ct | |
| --- | --- | --- | --- | --- |
|  | MammU6-4395470 | | 24.78 | |
|  | hsa-miR-135a*-4395343 | | 26.93 | |
|  | hsa-miR-923-4395264 | | 27.029 | |
|  | hsa-miR-188-5p-4395431 | | 30.167 | |
|  | hsa-miR-801-4395183 | | 30.975 | |
|  | hsa-miR-760-4395439 | | 32.43 | |
|  | hsa-miR-30e-4395334 | | 32.631 | |
|  | RNU48-4373383 | | 32.718 | |
|  | hsa-miR-30e*-4373057 | | 32.783 | |
|  | hsa-miR-625*-4395543 | | 33.309 | |
|  | hsa-miR-30a-4373061 | | 33.4 | |
|  | hsa-miR-378-4395354 | | 33.594 | |
| hsa-miR-630-4380970 | | 33.595 | |  |
| hsa-miR-93*-4395250 | | 33.6 | |  |
| hsa-miR-877-4395402 | | 33.733 | |  |
| hsa-miR-509-3p-4395347 | | 33.821 | |  |
| hsa-miR-126*-4373269 | | 33.967 | |  |
| hsa-miR-30d-4373059 | | 34.848 | |  |
| hsa-miR-425*-4395413 | | 35.012 | |  |
| hsa-miR-766-4395177 | | 35.393 | |  |
| hsa-miR-768-3p-4395188 | | 35.953 | |  |
| hsa-miR-335*-4395296 | | 36.144 | |  |
| hsa-miR-650-4381006 | | 36.569 | |  |
| hsa-miR-571-4381016 | | 36.87 | |  |
| hsa-miR-144*-4395259 | | 36.953 | |  |
| hsa-miR-30a*-4373062 | | 37.389 | |  |
| hsa-miR-409-3p-4395443 | | 38.638 | |  |
| hsa-miR-623-4386740 | | 38.649 | |  |
| hsa-miR-99b*-4395307 | | 39.613 | |  |
| hsa-miR-565-4380942 | | 39.671 | |  |
